# Single-cell genomic analysis of head and neck squamous cell carcinoma

**DOI:** 10.18632/oncotarget.18021

**Published:** 2017-05-19

**Authors:** Andres Stucky, Parish P. Sedghizadeh, Susan Mahabady, Xuelian Chen, Cheng Zhang, Gang Zhang, Xi Zhang, Jiang F. Zhong

**Affiliations:** ^1^ Division of Periodontology, Diagnostic Sciences and Dental Hygiene, and Division of Biomedical Sciences, Herman Ostrow School of Dentistry, University of Southern California, Los Angeles, CA, USA; ^2^ Department of Hematology and Blood Transfusion, Xinqiao Hospital, Third Military Medical University, Chongqing, P. R. China; ^3^ Department of Oral and Maxillofacial Surgery, Xinqiao Hospital, Third Military Medical University, Chongqing, P. R. China

**Keywords:** head and neck cancer, squamous cell carcinoma, HNSCC, single-cell genomics, circulating tumor cells

## Abstract

Head and neck squamous cell carcinoma (HNSCC) incidence or rates have increased dramatically recently with little improvement in patient outcomes. There is an unmet need in HNSCC to develop reliable molecular markers capable of evaluating patient risks and advising treatments. This review focuses on recent developments in single-cell molecular analysis of cancer, and its applications for HNSCC diagnosis and treatments. For proof of concept, we examined gene expression levels of 62 patients with HNSCC, and correlate the gene expression profiles to single-cell gene expression profiles obtained from a pilot single-cell study of CCR5-positive breast carcinoma cells. The single-cell molecular analyses complemented the lysate data and reveals heterogeneity of oncogenesis pathways with the cancer cell population. Our single-cell molecular analysis indicated that molecular heterogeneity exists in HNSCC and should be addressed in treatment strategy of HNSCC. Single-cell molecular technology can have significant impact on diagnosis, therapeutic decision making, and prognosis of HNSCC.

## MOLECULAR ANALYSIS OF HEAD AND NECK SQUAMOUS CELL CARCINOMA

Head and neck squamous cell carcinoma (HNSCC) is associated with high morbidity and mortality [1]. Although early stage HNSCC can be cured with radiation alone, entirely localized cancers are only detected in 30% of patients at the time of diagnosis, and the remaining 70% suffer some degree of recurrence or metastasis during their lifetime [2]. Furthermore, 61% of patients with advanced HNSCC experience local relapse, and up to 26% go on to develop distant metastases [3]. In many of these HNSCC cases, regional recurrence of the cancer and distant metastasis are the primary factors for poor prognosis, and thus the principal cause of death [3–5]. Patients with HNSCC also have a one in three likelihood of mortality from competing causes, most commonly lung cancer or cardiovascular disease [6]. Despite the expansion in our therapeutic repertoire for HNSCC management, mortality rates in recent decades have not significantly improved, and overall this cancer is understudied [7]. Although tobacco and alcohol use are the primary risk factors for developing HNSCC, human papillomavirus (HPV) has also recently been established as a significant risk factor for oropharyngeal HNSCC and is responsible for the growing prevalence of such cancers worldwide [8]. Diagnosis and treatment of HNSCC based on its molecular characteristics can improve the clinical outcomes.

However, conventional molecular assays previously used to study cancer gene expression and chemotherapeutic responses are done on whole cell populations, and therefore average the differences between individual cells. This approach grossly oversimplifies the various genetic profiles present in the tumor microenvironment (TME) and returns misleading results on the proportion and identity of cancer stem cells responsible for metastasis [9, 10]. In contrast, single-cell genomic profiling does not rely on pooled samples or cell populations, thus allowing for a higher fidelity representation of cell heterogeneity in the TME. As a result, single-cell sequencing allows each unique cell type within a system to be identified and analyzed.

The goal of this review is to illustrate the significant clinical potential of single-cell analysis of circulating tumor cells (CTCs). Although this technology is still in its infancy, it is clear that the integrated and simultaneous measurement of genome, transcriptome, and epigenome of a single cell in an integrated microfluidic device has the ability to realize a multidimensional understanding of heterogeneity, stratification, and cancer regulating phenotypes. Single cell genomics could potentially revolutionize the way we diagnose, treat, and prognosticate HNSCC.

There is currently an unmet need for research on single-cell molecular profiling in HNSCC in order to inform clinical parameters such as diagnostics, prognostics, and therapeutics. For other major carcinomas such as breast [11, 12], prostate [13], colon [14, 15], and lung cancers [16] among others, single-cell profiling has been applied both in cell lines and clinical samples. This application has led to knowledge of patient outcomes in the clinical setting, providing significant translational value. However, this has not been performed in HNSCC given the nature of this relatively recent technology involving single-cell profiling and the understudied nature of this disease. The analysis of single tumor cells overcomes the shortcomings of cancer heterogeneity and may aid in pinpointing driver mutations that result in the initial onset of tumor development, or identifying which mutations lead to metastasis, tumor progression, and resistance to therapy [17].

### Single cell isolation

There are three traditional approaches for single-cell isolation: micromanipulation (MM), laser capture microdissection (LCM), and flow cytometry (FC).

### Micromanipulation (MM)

Cell picking platforms usually consist of an inverted microscope combined with mechanically controlled micro-pipettes. This method is platform operator dependent, and setup involves cell suspension in a dish or 96-well plate. Cells are then individually selected, and transferred to a collection tube with the micro-pipette. An advantage of micromanipulators is that they allow the precise separation of living cells, and may even support the capture and separation of some prokaryotic cells [18] for further downstream analysis. Disadvantages of micromanipulation, however, are extremely low capture throughputs, stress from mechanical manipulation, and high variability.

### Laser capture microdissection (LCM)

LCM is a cell isolation technology mainly used to extract sections from solid tissue [19, 20]. Once a cell or tissue section has been identified under a microscope, the desired sections can be specified by the operator on an interactive display, and laser-cut along the specified trajectory. In the case of a gravity-assisted microdissection, such as with the Leica LMD7000^®^, dissection takes place on an inverse mounted microscope and sections fall underneath onto a collection plate. In other cases, an adhesive substrate can be used to recover and relocate the dissected tissue section. Laser pressure catapulting (LPC), involving a brief defocused laser pulse, excites local plasma below the cut cell for propelling it vertically into a nearby collector container [21, 22]. Although some LCM systems enable the extraction of live cells for culture and analysis, most LCM systems produce samples that are cryo-fixed or embedded in paraffin for staining and histological purposes [23].

### Flow cytometry (FC)

Flow cytometry is a cell sorting technique that relies on the strict discrimination of expressed surface proteins. In FACS, suspended cells are pressure-driven through a flow cell and lined up, then optically excited by a laser beam and interpreted by downstream optical detectors that capture cell specific signals. The intrinsic cell-specific physical, chemical, or optical properties are further enriched with conjugated fluorescent dyes targeting specific cell surface markers. This results in size fractionation and counting, as well as cell sorting. In FACS, a flow of cells are made into a continuous stream of droplets. Electrically charged plates are used to deflect and guide the cell-containing droplets to collector microcells. FACS is currently one of the most popular platforms for cell sorting, although high costs of flow cytometers results in limited use [24]. Moreover, sorted cell viability may be compromised by non-specific fluorescent molecules and rapid cell flow of the cytometer. Also cells are subjected to laser stimulation during FACS analysis, which can cause cell damage and invalidate the experiment [25].

### Microfluidics for single-cell isolation

Microfluidics is a recently developed single-cell isolation technique with micrometer diameter channels [26, 27]. More sophisticated and advanced microfluidics devices have well control valves, pumps and take advantages of laser technology such as these developed in our laboratory [10, 17, 28, 29]. Microfluidic devices can also be applied to culture single cells in microfluidic chambers [30], where they are sequentially treated with specific factors and lysed for downstream sequencing [31]. Given that viable living cells are mainly encountered in liquid environments, microfluidics coupled with high-definition immunosorting has rapidly become the preferred tool for single-cell isolation [32, 33]. These systems are versatile enough to manipulate volumes ranging from microliters to femtoliters, and sensitive enough to handle individual cell sizes averaging between 500-micron diameter in some eukaryotes to a few nanometers for prokaryote cells. Cells can be arrested in nanometer droplets for visualization or microchemical reactions using capillary gates on microchannels embedded in a microfluidic chip.

The materials used to fabricate microfluidic chips have also rapidly evolved. Options include glass and silicone chips, which have ideal capillary properties, but are expensive to fabricate; others consist of plastics, thermosets, and elastomers, which allow for low cost prototyping and high-density integration of valves and microchannel gating. Hydrogel materials, on the other hand, are highly permeable and enable single-cell cultures. Most microfluidic chips are now made of polydimethylsiloxane (PDMS), an elastomer that has gained popularity due to its biocompatible characteristics, gas permeability, and low fabrication cost [34]. This enables high throughput, cost efficient, portable systems, with more powerful microscale analytical capabilities [35]. Among the various types of microfluidic systems, three main principles are used to isolate single cells. In oil droplet-based microfluidic systems, single cells can be contained and thereby isolated within aqueous drops, suspended in a network of oil-filled channels [10]. This system allows for capture, barcoding, and amplification of the entire mRNA transcriptome of individual cells, and provides a robust output of up to several thousand single-cells per second [36, 37]. Other systems are made up of pneumatic membrane valves with pressurized air to digitally open or close gating valves in order to deflect cells through a microfluidic downstream network of channels. This valve-based approach usually requires an operator to isolate individual cells. When compared to oil droplet technologies, pneumatic membrane valve systems provide limited throughput yet very high sensitivity.

Microvalve platforms consist of passive flow microchannels, allowing only one cell to enter the “trap”. Adjusting the trap size to the size of the average cell in the sample minimizes double occupation. By using various size traps, these systems can analyze a large number of individual cells in parallel [10, 36]. Hydrodynamic trapping can also be integrated into handheld pipettes to enable manual single-cell pipetting without the need for micromanipulation under microscopy. Integrating microfluidic technologies with flow cytometers have also been proposed in order to miniaturize large and complicated flow cytometer functions, such as cell sorting and counting, to small and affordable devices [38, 39]. These microfluidic lab-on-a-chip technologies for single-cell applications offer exciting possibilities, especially for the study and catalogue of stem cells and cancer cells.

### Circulating tumor cell (CTC) technologies

CTCs are cells that have migrated from the primary tumor into the bloodstream. These cells travel in the circulatory system, then deposit in a distant organ and proliferate at the new location resulting in metastatic disease. Therefore, molecular characterization of CTCs provides a non-invasive and real-time molecular profile of the tumor burden for prognosis as well as treatment evaluation. Currently, CTC enumeration (CellSearch) is an FDA approved assay for prognosis of metastatic breast, prostate, and colon cancer [40–43]. This method takes advantage of tumor epithelial cell expression of EpCAM, KRT 8, 18, and 19, and the absence of leukocyte marker CD45, to identify and isolate circulating cancer cells. This platform was used in a large cohort study to indicate that CTCs are exceptionally rare in healthy subjects and patients with nonmalignant diseases, but present in a range of frequencies in various metastatic carcinomas depending on the origin and severity of disease [40]. This system was also used in a series of studies to monitor CTC flow of patients with noticeable metastatic cancer during the course of treatment. These studies demonstrated that CTC enumeration can be a reliable indicator of patient prognosis in lung [44] and pancreatic cancer [45], and an independent predictor of progression-free survival and overall survival in patients with prostate, breast, and colorectal cancers [42, 46].

The clinical implications of CTC enumeration (CellSearch) may be limited due to lack of sensitivity, and captured circulating epithelial cells may not be malignant in many cases [47, 48]. Single-cell sequencing directly promotes a new generation of genomic medicine as high resolution single-cell genomics reveals the complexity within cell networks responsible for cell identity and function. In addition to immunological enumeration, a simple genetic screen will allow rapid detection of large abnormalities such as aneuploidy, single-gene disorders, and chromosomal translocation. Consequently, a rapid increase in the available single-cell data is certain to accelerate the cataloging of TME, and propel the identification of cancer specific biomarkers for diagnosis and targeted therapies.

Single-cell genomics has already demonstrated revolutionary potential by revealing a diversity of concurrent molecular paths critical for cancer function [9, 10, 14, 15], The advantages of a systematic monitoring of tumor cell circulation are multiplied by an extensive genomic record of CTC heterogeneity. This will transform the way we approach personalized medicine for HNSCC and significantly mitigate the impact that therapies have on patient's quality of life. High-definition representation of the ‘basic cancer molecular cascades’ will add an additional layer to our biological understanding of cancer progression, most significantly for CTC technologies, by the identification of novel cell membrane markers for improved enrichment of otherwise undetected cancer cells.

A study by Guney et al. in 2007 evaluated the clinical relevance of blood-borne cancer cells for patients with HNSCC before surgery. This study demonstrated the detection of CTC in one-third of patients using epithelial antibody-bound magnetic beads, and concluded that patients with stage III and IV tumors were 5 times more likely to show CTCs compared to those with stage I or II tumors. Furthermore, they indicated that 2 out of 7 patients who presented CTCs had local recurrences during follow up, but none of the CTC negative-group relapsed [49]. Another study featuring patients with locally advanced HNSCC identified clinical factors (tumor, nodal, and lymph metastasis) that were predictive of CTC presence in the circulatory system via CellSearch. They state that CTCs were identified in 43% of patients, with increasing susceptibility as clinical parameters increased [50]. In another study, the highest incidence CTC was found in hypopharyngeal cancer (40%), followed by oropharyngeal cancer (13%), and none detected in oral cavity cancer [51]. CTCs were also identified in 67% of the oral cancer patients surveyed, followed by oropharyngeal cancer with 47% of patients [52]. These studies indicated that CTC can be used clinically to predict and improve clinical outcomes of HNSCC. However, despite several promising results, the clinical impact of CTC assay in HNSCC remains unclear primarily due to lack of molecular characterization and clinical study in HNSCC.

Squamous cell carcinomas are characterized by very specific expression patterns for several keratins [53], and several serum tumor markers have been evaluated for their predictive value in HNSCC prognosis. However, the success of this approach has been limited, due to poor assay sensitivity and lack of receptor characterization [54, 55]. One approach with relative success has used negative leukocyte depletion for cancer cell enrichment in patients with HNSCC and colorectal cancer. This study revealed two major cell populations, EpCAM+CD45− and EpCAM−CD45−; and numbers for both cell types was significantly increased in cancer patients when compared to these in the healthy controls [56]. Numbers of both cells types substantially decreased in HNSCC patients following chemotherapy [56], but CTC populations were found to be more heterogeneous than initially believed as another study identified cells with unique stem (CD133+), epithelial (cytokeratin+), and mesenchymal (N-cadherin+) characteristics, as well as epithelial-mesenchymal (cytokeratin+, N-cadherin+) positive cells, and epithelial cells with stem cell-like characteristics [52]. Patients without detectable CTCs are more likely to have longer disease-free survival times when compared to CTC-positive patients [57]. The major obstacles to CTC classification are now being overcome by the increased availability of modern sequencing technologies coupled with integrated microfluidic chip devices for gene expression profiling.

### Single-cell molecular analysis of HNSCC

Gene expression is an important aspect of both normal and pathological development of cells. Microfluidic technologies have demonstrated to be ideal for extracting total mRNA from single cells with minimal loss. cDNA synthesis can be performed on the same chip for high efficiency single-cell gene expression profiling, and reduce minimum detectable number of mRNA molecules [29, 58–60]. This approach results in a reliable and straight-forward system suited for general use in most research laboratories, as well as a spectrum of clinical assays. However, first generation technologies, such as fluorescent cell sorters and robotic micropipettes, have prohibitive costs and technical difficulties that substantially limit their availability for research and medical applications. Furthermore, handling the components of a single-cell system with current microliter scale tools inevitably leads to inaccuracy and major material loss, discouraging their use for large-scale standardized applications. Therefore, the development of a simple and inexpensive integrated microfluidic device to manipulate nanoliter fluid volumes consistently and accurately represents an ideal tool for single-cell analysis.

Some of the most promising applications for single-cell technology include the early detection and characterization of HNSCC, as well as determining if intervention should be surgical, chemical, or multimodal, which is crucial to avoid unnecessary surgery in cases where larynx (voice) preservation is a priority. Further, single-cell screening will help prevent overtreatment in some cases of intense multimodal therapies, which can cause serious organ impairment due to radiation toxicity, and are only successful in 30% of patients [61]. Single-cell fingerprinting can generate a virtual map of the CTC population and trace the clonal evolution of seeding cancer stem cells. Single-cell profiling can also inform the most appropriate targeted chemotherapy and predict patient outcomes, which have already been demonstrated for several other cancers, but not for HNSCC [17, 62]. Therefore genetic mutations within a cell can interact with each other, genetic alternations within a cell could reveal whether a specific pathway is altered by these mutations. An example is provided in this review from our study of HNSCC in the next section.

When selecting a chemical or biological agent, RNA sequencing from CTCs demonstrates gene expression patterns of the cancer, and can be used to determine the most appropriate course of treatment on an individual basis. For instance, identification of imatinib-sensitive KIT mutations in sinonasal carcinomas, a series of rare tumor type refractory to multimodal treatment and lacking effective systemic therapies, may suggest specific targeted therapy [63]. Single-cell genomics can also screen for HPV-related and non HPV-related HNSCC. Appropriate genetic screening can aid in distinguishing lesions that appear benign upon histopathologically examination, but actually represent cancerous lesions [64]. Gene fusions in HPV-positive oropharyngeal carcinoma are associated with significant up-regulation of 16 genes including EGFR and ERBB4, and down-regulation of PTPRT, ZNF750, DLG2, and SLCO5A1. This gene expression profile can clearly justify a specific course of action clinically [65]. Studies have shown that HPV-positive and HPV-negative HNSCC differed significantly in the activity levels of key transcription factors such as AP1, STATs, NF-κB, and P53, and this particular genotype can be effectively treated with combinations of anti-NF-κB and anti-STAT therapies [66]. The study of single CTCs promises to dramatically transform the application of genomic technologies in cancer prognosis and treatment.

### Molecular characteristics of HNSCC

Definitive HNSCC diagnosis presently relies on histopathological examination of biopsy tissue. In some cases, diagnosis can be challenging due to overlap of features with other carcinomas; for example, metastatic tumors to the head and neck that are not primary HNSCC. Another factor in diagnosis is subjectivity of the pathologist, and well-differentiated cancers may be mistaken for benign lesions instead of malignancy, in some cases. Molecular diagnostics is not currently available for HNSCC despite report of gene amplification of HNSCC [67]. Treatment of HNSCC involves surgery and/ or chemo-radiation depending on clinicopathologic parameters, such as disease stage. Surgical treatment of the head and neck is complicated by the sensitive nature of these anatomic structures, and the need to provide the best quality of life for the patient. Thus, a thorough survey and revision of HNSCC-specific molecular targets can dramatically accelerate the clinical implementation of CTC genomic marker screening. However, the accurate and reliable stratification of HNSCC for outcome prediction has been challenging as conventional approaches have proven inadequate in predicting responses to nonsurgical cancer therapy [68]. In cases where a patient undergoes surgical resection of the tumor, histologic examination of the tumor and regional lymph nodes is still the best way to assess the pathologic stage of a patient (pTNM), and verify the initial diagnosis and inform proper adjuvant therapy. Occasionally, assigned stage can vary due to differences in staging methods or method sensitivity, rather than the actual extent of the disease [67–70]. Furthermore, comparisons between cTNM and pTNM staging often render inconsistencies due to varying staging methods. All things considered, only one-third of these carcinomas are detected in due time [71]; and despite great developments in diagnosis and therapies, the most reliable curative treatment is surgical tumor resection, and the best prognostic indicator of relapse is histological confirmation of lymph node metastasis [72]. Therefore, molecular characteristics of HNSCC could significantly improve current practices in diagnosis and therapy of HNSCC [73].

### Potential of single-cell molecular analysis for HNSCC

Targeted molecular therapies are rapidly becoming a reality as drug trials now focus on biomarker screenings to select patients who will benefit most from targeted therapies [74], including EGFR, VEGF, NF-κB, and platinum-based therapies. However, progress in development of this therapy has been delayed. Single target therapies, such as MYC and EGFR, are not as effective as initially expected, such as the use of EGFR inhibitor monotherapies [75, 76] due to the heterogeneity involved in tumorigenesis. Current strategies now seek to combine several EGFR inhibition or complementary therapies by targeting non-overlapping and convergent signaling pathways. Resistance to EGFR inhibiting is generally overcome by the simultaneous use of EGFR and VEGF inhibitors [77]. An equally problematic situation is targeting the MYC genetic programs, which are known to orchestrate cell proliferation, metabolism, and stress responses.

We examined gene expression of 62 patients with various degrees of HNSCC (Gdc-portal.nci.nih.gov), and correlate with results obtained from a pilot single-cell screening of CCR5-positive breast carcinoma cells. Pathway enrichment analysis (IPA^®^) identified robust expression of many genes involved in several well-known oncogenic pathways. MYC transcription was predicted to have the greatest number of upregulated genes in the data set (*p*-value 2.55E-87), followed by HNF4α (*p*-value 1.14E-77), and TGFβ1 (*p*-value. 4.22E-66); all of which are important regulators of cancer progression and growth. Single-cell molecular analyses complemented the lysate data, and reveals heterogeneity of these oncogenesis pathways.

**Figure 1:**
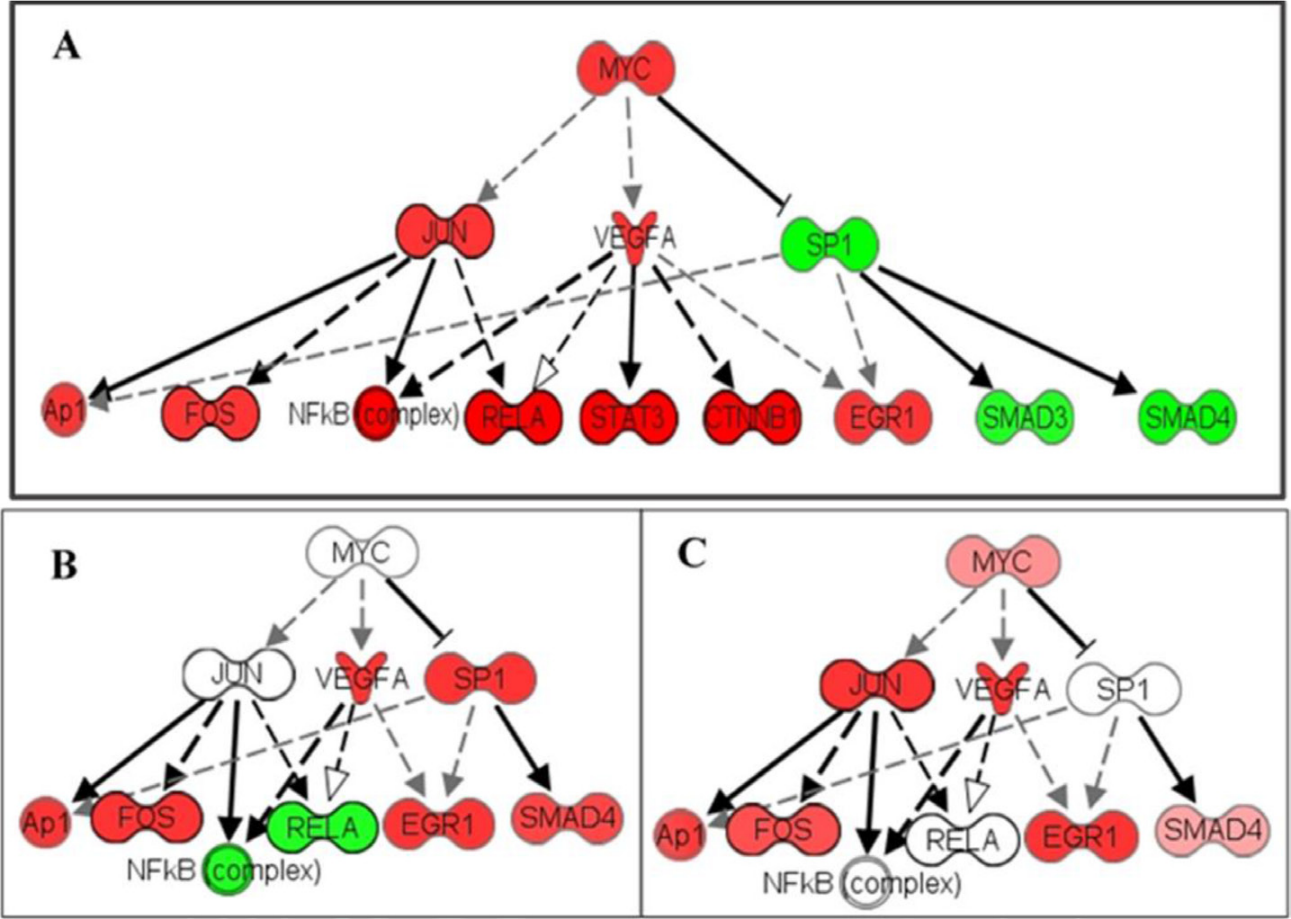
Heterogeneity of MYC pathway activation in cancer The MYC pathway is activated in a patient with HNSCC by analysis of the cell lysate. MYC pathway activation was also detected in single CCR5+ breast cancer cells. (**A**) RNA-seq of a HNSCC lysate shows activation of multiple members in the MYC pathway. (**B**) Single-cell RNA-seq shows activation of MYC members through VEGFA and SP1. (**C**) Another single-cell RNA-seq shows activation of the MYC pathway through JUN and VEGFA. This heterogeneity of MYC pathway activation could explain why target therapies based on cell lysate results are often not effective or fail to control the cancers which contain multiple activation patterns of the MYC pathway. Red: upregulation; Green: down-regulation. Up- or down-regulation are calculated with fold difference of expression level cancer lysate/non-cancer lysate or cancer single cell/normal single-cell respectively. Darker color indicates higher fold changes.

MYC induced overexpression and/or rearrangement has been broadly studied in certain hematopoietic tumors including leukemia [78], lymphoma [79], and Burkitt lymphoma [80], but the connection between MYC transcription and HNSCC is not yet well understood. Literature in this field suggests that targeting upstream binding elements interacting with MYC repressors can potentially suppress the progression of certain forms of head and neck cancers [81]. Our data demonstrates a strong associated downstream MYC signal in the 62 HNSCCs, and high gene expression values for all MYC, JUN and VEGFA downstream molecules. When these expression values of whole cell lysates from HNSCC were compared with a small cohort of captured single CCR5-positive breast cancer cells, it is evident that individual cells express only a fraction of the upregulated genes in the cell lysates (Figure 1). Associated genes are expressed in a heterogeneous manner, and activated genes in the signaling cascade can vary for every cell. Accordingly, they will not share an identical response to the same pharmacological therapy; and in the absence of patently conspicuous gene mutations, whole lysate sequencing will contribute minimally (if at all) to therapeutic decision making.

In general, pathways that converge on MYC confer a selective advantage to cancer cells by adapting the metabolism of proteins, lipids, carbohydrates, and nucleic acids in the TME [82]. MYC signaling can affect cell behavior by several important downstream effector molecules, which can produce diverse outcomes depending on cellular context. Downstream effectors may include PI3K–AKT–, mTOR, RAS–MAPK, and NF-κB, and consequently it can be difficult to determine the appropriate molecular inhibitor to prescribe [83]. In the HNSCC data set, high levels of VEGF, RELA, and NF-κB expression values suggest that targeting this pathway is a reasonable course of treatment, since NF-κB. However, when we observe the same expression in our single-cell data, we see that NF-κB is not expressed in all cells, and a different target may be more beneficial (Figure 1). Similar to EGFR, as described previously, selection of a single molecule inhibitor is not sufficient in most cases. Targeting high-expressing MYC tumor cells and dual molecule inhibitors of RPI3K and BRD4 therapies seem to indicate increased inhibition of MYC [84]. Dual histone deacetylases (HDAC) and phosphoinositide 3-kinase inhibitors are used against B-cell lymphoma MYC hyperactivation [79]. Our data stresses the importance of appropriately identifying targeted chemotherapy for the specific cancer stem cell clones, or CTCs detected in the bloodstream, which are most likely seeds of metastasis.

MYC-initiated transcription can also increase JUN expression and recruitment to the cell nucleus [85] triggering inhibition of p53 activity [86]. Tumor cells overexpressing MYC reprogram their metabolism to depend on glutamine for the continuance of cell viability. Tumor cells increasingly rely on glutamine for their bioenergetic and metabolic needs, triggering increased cellular dependency to glutamine and an increased susceptibility to glutamine deprivation, a phenomenon known as the Warburg effect [82, 87]. Although the relationship between amplification and overexpression is not yet clear, MYC pathway upregulation has been significantly correlated with aggressive tumor phenotypes and poor clinical outcomes [88]. Identification of molecular biomarkers for guiding therapy without fully understanding intratumoral heterogeneity is misleading, and identified biomarkers can be premature thus subjecting patients to unnecessary risks.

### Conclusion and perspective

Until very recently, it has been common practice to compare data from a single tumor source to samples of healthy or control tissue. This approach has caused HNSCC to be largely regarded as a complex collection of cancer cells with little variation among each other (clonal subpopulations), and this idea has nurtured a one-size-fits-all attitude toward HNSCC patient management [73]. Today, molecular genetic analysis reveals not only individual tumor differences, but also large-scale alterations that permit the identification of vital cancer subtypes, such as HPV-positive HNSCCs. Prior to single-cell technology; studies identified a vast number of genetic mutations in samples from patients with HNSCC [89, 90]. Yet a satisfactory representation of intra-tumor heterogeneity has not been possible; and multi-site tumor sampling continues to use conventional lysate sequencing by averaging genetic differences among clones, which inevitably assumes a generalized view of all cells in the tumor. Nevertheless, tumor ‘field cancerization’ is helping explain local changes in HNSCCs. Moreover, a deeper appreciation of intra-tumoral differences is critical in developing treatment strategies aimed at increasing tumor-specific antigen responses, all while targeting critical components of the genetic makeup that supports tumor development and growth [73].

In this review, we demonstrate that single-cell sequencing of tumor cells may provide a virtual snapshot of tumor heterogeneity, and real-time assessment of significant molecular vectors are most likely to be successful therapeutic targets. Considering the many technical advantages of high resolution microfluidic sorting and the great wealth of information that can be extracted by the systematic genomic sequencing of single circulating cancer cells, this approach is likely to become the standard practice in the prognosis and treatment of HNSCC. The currently poor outcomes in advanced HNSCC cases supports the unmet need for research on single-cell molecular profiling to inform clinical parameters such as diagnostics, therapeutics, and prognostics as has been achieved with other carcinomas.
